# Comparative study of microbial community from mining wastes - focus on future recovery of copper

**DOI:** 10.1186/1753-6561-8-S4-P182

**Published:** 2014-10-01

**Authors:** Ingrid Regina Avanzi, Louise Hase Gracioso, Marcela Galluzzi dos Passos Baltazar, Marcela Nunes Veiga, Luciana Jandelli Gimenes, Elen Aquino Perpetuo, Claudio Augusto Oller do Nascimento

**Affiliations:** 1 Center for Environmental Research and Training, CEPEMA-POLI-USP, SP, Brazil

## Background

The interaction between microorganisms and heavy metals has been occurring since the beginning of life on the planet, 4 billion years ago, which allowed the biological evolution of resistance in systems and the survival of these microorganisms in environments containing high metals concentrations of metals [1]. Recently, a bioremediation review presented by Perpetuo *et.al*. [2] considers the bioremediation technique as a feasible alternative for treatment and recovery of sites contaminated by heavy metals. However, a previous study of the microbial community living in these areas is necessary as well as the isolation of resistant and effective microorganisms with characteristics favorable to bioprocess, for remotion or concentration of these metals and also for subsequent metal reuse. This study investigates the bioprospection of natural selected copper-resistant organisms from a copper mining located in Pará, Brazil, for future reuse purposes.

## Methods

Microorganisms were isolated from mining wastes by culture enrichment technique; this procedure was repeated 4 times. The isolates were inoculated into MJS medium containing different concentrations of chloride copper (1mM, 2.5mM, 5mM, 7.5mM and 10 mM) and incubated in plates for 72 h at 30 ºC. Cellular growth and residual copper from the culture medium were monitored by spectrophotometer UV-Vis (600 nm) and inductively coupled plasma optical emission spectrometry (ICP-OES), respectively. Strains identification was performed by mass spectrometry (MALDI-TOF). This technique produces a protein spectrum of each sample and then compares it to a Biotyper database (Bruker Daltonics), confirming usual molecular identification.

## Results

Biodiversity was determined by monitoring cultivable bacterial morphotypes. This biodiversity study intended to correlate the number of different species and their localization within the analyzed micro-environments. Until the conclusion of this article, the predominant species found was *Pseudomonas aeruginosa *(12 of 19 isolated strains), followed by *Bacillus cereus *(3 strains) and *Burkholderia cepacia *(1 strain). These species were found in all the samples collected each 3 months and are known in literature as resistant to heavy metals. Experiments containing high concentration of copper were carried on and the high resistance (5mM of copper) was confirmed. Hussein *et.al*. [3] isolated *Pseudomonas *sp. strains from a sewage treatment plant; with potential tolerance to copper, nickel, zinc, cadmium and chromium. Like in this study, Castro-Silva [4] also related strains of Bacillus resistant to heavy metals, but in a copper mine located in Santa Catarina, Brazil; and *Burkholderia *sp. has been described in the literature as symbionts of plants for the bioaccumulation of heavy metals [5]. Once we have verified cellular growth in the presence of significant metal concentrations and high capacity for metal biosorption under aerobic conditions, these bacteria can potentially be applied to *in situ *bioremediation of aqueous systems contaminated by heavy metals, also allowing for the recovery of these metals.

## Conclusion

This work has great importance due to the low cost of systems treatment compared to conventional ones, allowing a better use of copper wastes and consequently better mining economic return. Moreover, the main advantage is further reduction of environmental impact caused by the mining activity.

## References

[B1] Siver S Phung LT A bacterial view of the periodic table: genes and proteins for toxic inorganic ions J Ind Microbiol Biotechnol 2005 32 11-12 587 605 10.1007/s10295-005-0019-6 16133099

[B2] Perpetuo EA Souza CB Nascimento CAO Capri A Engineering Bacteria for Bioremediation Progress in Molecular and Environmental Bioengineering - From Analysis and Modeling to Technology Applications InTech 2011

[B3] Hussein H Ibrahim SF Kandeel K Moawad H Biosorption of heavy metals from waste water using Pseudomonas sp. Electron J Biotechnol 2004 7 1

[B4] Castro-Silva MA Heavy metal resistance of microorganisms isolated from coal mining environments of Santa Catarina Braz J Microbiol 2003

[B5] Huang GH Tian HH Liu HY Fan XW Liang Y Li YZ Characterization of Plant-Growth-Promoting Effects and Concurrent Promotion of Heavy Metal Accumulation in the Tissues of the Plants Grown in The Polluted Soil by Burkholderia Strain LD-11 International Journal of Phytoremediation 2013 15 10 991 1009 10.1080/15226514.2012.751354 23819291

